# 

**Published:** 1896-10

**Authors:** 


### TABLE OF CONTENTS.

ORIGINAL COMMUNICATIONS. PAGE

Salivary Calculi. By GUSTAV FÜTERER, M.D. . . . . . . . . . . . . . . 621
Directly Connected Static Machines. By WILLIAM ROLLINS, Boston, Mass. . . . . . . . . . . . . . . . . . . . . . . . . . . . . . . . 635
The Application of the Principle of Crown-Work to Metal Plates. By JOSEPH HEAD, M.D., D.D.S., Philadelphia . . . . . . . . . . . . . . . 637
What can we demonstrate? By DR. G. A. MILLS, New York . . . . . . 639
Presidential Address of Dr. A. V. Elliott, Florence, Italy . . . . . . . . 640

ABSTRACTS AND TRANSLATIONS.

Some Points in Connection with the Bacteria of the Mouth. By J. W. WASHBOURN, M.D., F.R.C.P., and K. W. GOADBY . . . . . . . . . . . 642

REPORTS OF SOCIETY MEETINGS.

National Association of Dental Faculties . . . . . . . . . . . . . . . . . 652
American Dental Association . . . . . . . . . . . . . . . . . . . . . . . 659
Odontological Society of Pennsylvania . . . . . . . . . . . . . . . . . . 667

EDITORIAL.

Is Reorganization Necessary? . . . . . . . . . . . . . . . . . . . . . . 674

DOMESTIC CORRESPONDENCE.

The History of Cataphoresis corrected . . . . . . . . . . . . . . . . . 678

BIBLIOGRAPHY . . . . . . . . . . . . . . . . . . . . . . . . . . . . . 680

NOTES AND COMMENTS . . . . . . . . . . . . . . . . . . . . . . . . 681

CURRENT NEWS.

Northeastern Dental Association . . . . . . . . . . . . . . . . . . . . 683

SELECTIONS.

Fatal Acute Poisoning by Cocaine . . . . . . . . . . . . . . . . . . . . 683
Formula for Local Anæsthesia . . . . . . . . . . . . . . . . . . . . . . 684
The Medical Profession in the United States . . . . . . . . . . . . . . 684

